# Time Series Electrical Motor Drives Forecasting Based on Simulation Modeling and Bidirectional Long-Short Term Memory

**DOI:** 10.3390/s23177647

**Published:** 2023-09-04

**Authors:** Thi-Thu-Huong Le, Yustus Eko Oktian, Uk Jo, Howon Kim

**Affiliations:** 1 Blockchain Platform Research Center, Pusan National University, Busan 609735, Republic of Korea; yustus@islab.re.kr; 2 IoT Research Center, Pusan National University, Busan 609735, Republic of Korea; 3 School of Computer Science and Engineering, Pusan National University, Busan 609735, Republic of Korea; jouk@islab.re.kr

**Keywords:** Bi-LSTM, deep learning, FFT, frequency domain, signal processing, simulation modeling, three-phase DTC induction motor, time series forecasting

## Abstract

Accurately forecasting electrical signals from three-phase Direct Torque Control (DTC) induction motors is crucial for achieving optimal motor performance and effective condition monitoring. However, the intricate nature of multiple DTC induction motors and the variability in operational conditions present significant challenges for conventional prediction methodologies. To address these obstacles, we propose an innovative solution that leverages the Fast Fourier Transform (FFT) to preprocess simulation data from electrical motors. A Bidirectional Long Short-Term Memory (Bi-LSTM) network then uses this altered data to forecast processed motor signals. Our proposed approach is thoroughly examined using a comparative examination of cutting-edge forecasting models such as the Recurrent Neural Network (RNN), Long Short-Term Memory (LSTM), and Gated Recurrent Unit (GRU). This rigorous comparison underscores the remarkable efficacy of our approach in elevating the precision and reliability of forecasts for induction motor signals. The results unequivocally establish the superiority of our method across stator and rotor current testing data, as evidenced by Mean Absolute Error (MAE) average results of 92.6864 and 93.8802 for stator and rotor current data, respectively. Additionally, compared to alternative forecasting models, the Root Mean Square Error (RMSE) average results of 105.0636 and 85.7820 underscore reduced prediction loss.

## 1. Introduction

Three-phase DTC induction motors are widely utilized across industries, including electric vehicles, industrial automation, and ship propulsion systems, owing to their commendable efficiency and robustness [1]. Accurately predicting electrical signals within these motors, encompassing stator and rotor currents and voltages, is paramount for achieving optimal motor performance and ensuring effective condition monitoring. Despite the crucial significance of accurate signal forecasting, the complexity of DTC induction motors, particularly in ship propulsion systems, introduces notable challenges that require dedicated exploration, such as combination with varying operating conditions in different environments, which need to be improved for accurately predicting these signals using traditional methods by [2].

In shipboard environments, Direct Torque Control (DTC) induction motors are crucial for ensuring safe and efficient vessel operations. These motors are subjected to a diverse range of load conditions, varying sea states, and complex electromechanical interactions arising due to the dynamic nature of maritime operations [3]. Achieving precise predictions of electrical signals from these motors is paramount as it directly contributes to optimizing energy consumption, enhancing operational reliability, and facilitating timely maintenance interventions [4]. However, the inherent complexities of shipboard settings introduce additional layers of intricacy to the prediction task.

In this context, simulation modeling emerges as a crucial tool for comprehending and optimizing the behavior of DTC induction motors within shipboard environments. Simulation modeling has garnered prominence in electrical engineering, serving as a valuable instrument for conducting comprehensive tests, performing analyses, and optimizing electrical systems [5]. This approach offers a cost-effective and efficient means of generating multiple instances of DTC induction motors across varying operating conditions [6]. It serves to address the limitations that deep learning models may encounter when confronted with constrained or fluctuating training data and specific operational scenarios [7,8,9]. However, achieving accurate signal forecasting demands the development of models that intricately replicate the actual dynamics of the motor system. This requirement underscores the inclusion of multiple identical DTC induction motors within simulation models [10,11]. However, adopting a series of identical motors introduces challenges in distinguishing and discerning the distinct data associated with each motor.

Recent years have witnessed the emergence of advanced modeling techniques to enhance the precision of signal forecasting in induction motors. Statistical models, Artificial Neural Networks (ANNs), and Deep Learning (DL) models have garnered attention as potential solutions in this context, as emphasized by [12]. Notably, DL models such as recurrent neural networks (RNNs) and Convolutional Neural Networks (CNNs) demonstrate a remarkable ability to capture temporal dependencies and effectively handle intricate, high-dimensional input/output data [13]. Nevertheless, the challenges posed by shipboard DTC induction motors encompass multifaceted dimensions that extend beyond the inherent complexities of the motor system. These challenges intricately interplay with the specific dynamics of maritime operations and the imperative for accurate forecasting under varying and fluctuating operating conditions. As a result, these challenges remain an ongoing focal point for dedicated research efforts.

To tackle these intricate challenges effectively, this study introduces an innovative approach that amalgamates the transformed Fast Fourier Transform (FFT) with a Bidirectional Long Short-Term Memory (Bi-LSTM) network. By harnessing both techniques’ strengths, this approach offers precise and dependable predictions of electrical signals stemming from DTC induction motors in shipboard contexts. The adoption of the Bi-LSTM model stems from its prowess in adeptly capturing temporal dependencies—a critical attribute for ensuring accurate forecasts amid the dynamic conditions prevalent in maritime environments. Furthermore, the efficacy of the Bi-LSTM model extends beyond the boundaries of this research domain, finding relevance in a diverse array of applications. These include its successful deployment in predicting phenomena such as COVID-19 [14], forecasting water levels [15], and projecting wind speed and solar irradiance [16], as well as anticipating solar power production [17]. This extensive applicability underscores the versatility and potency of the Bi-LSTM model across various predictive tasks and domains.

The rest of the paper is organized as follows. Section 2 briefly reviews the literature on simulation modeling and forecasting of electrical signals in DTC induction motors. Section 3 describes the proposed methodology in detail, including the data collection and preprocessing, Bi-LSTM model architecture, and model training and evaluation. Section 4 presents the experimental results and discussion. Finally, Section 5 concludes the paper and suggests future research directions.

## 2. Related Work

Simulation modeling has emerged as a pivotal tool within the realm of electrical engineering, facilitating comprehensive testing, analysis, and optimization of intricate electrical systems. Le [18] aptly underscored the advantages of simulation modeling, emphasizing its cost-effectiveness and efficiency. This approach enables the thorough study of electrical systems without necessitating resource-intensive and time-consuming physical experiments. Furthermore, simulation modeling empowers researchers to generate multiple instances of DTC induction motors under varying operating conditions, thereby addressing the limitations that deep learning models might encounter when faced with constrained or fluctuating training data and specific operational scenarios, as highlighted by previous works [19,20,21,22]. Moreover, in the work of Ghimire et al. [23], a marine DC hybrid power system is introduced and formulated through a bond graph modeling approach. The study effectively showcases the capability of the developed system model to capture the fundamental dynamics inherent in real-world systems. In another recent investigation, Goolak et al. [24] delve into the realm of vehicle applications, presenting a mathematical modeling strategy for induction motors. Their study focuses on an induction motor featuring symmetrical windings, with simulations executed within the MATLAB programming environment.

Precise forecasting of electrical signals within DTC induction motors demands the creation of models that faithfully capture the dynamics exhibited by actual motors. It necessitates the incorporation of multiple identical DTC induction motors within simulation models to ensure a faithful reflection of real-world motor behavior. Scholars such as Grabowski et al. [25] and Lai et al. [26] have duly emphasized the significance of integrating multiple identical DTC induction motors within simulation models to capture system dynamics accurately.

The proficiency of DL models in capturing temporal dependencies and handling intricate high-dimensional data have demonstrated considerable promise. Studies such as [27,28] have illuminated the potential of Convolutional Neural Networks (CNNs) in modeling and forecasting time series data, as evidenced in load forecasting. Notably, Song et al. [29] introduced a bi-level LSTM (Long Short-Term Memory) prediction model for predicting a machine Remaining Useful Life (RUL). However, accurately predicting electrical signals of DTC induction motors using DL models presents challenges due to motors’ inherent nonlinearity, complexity, and varying operating conditions [30].

In addressing the intricacies of signal prediction, several approaches have proposed a transformation from the time domain to the frequency domain. This transformation has proven to be an effective solution in mitigating the difficulties inherent in signal prediction tasks. It enhances the representation of diverse input features, thus accentuating even subtle discrepancies among comparable motor signals. Notable research endeavors such as [31,32,33] have validated the efficacy of this transformation in elevating signal forecasting accuracy. Additionally, Toma et al. [34] proposed a hybrid model integrating Discrete Wavelet Transform and extreme learning machines to predict bearing fault classification in induction motors. Koh et al. [35] employed CNNs to predict rotors in DTC induction motors. However, more research is warranted, specifically utilizing effective transformation methodologies on simulation data, such as the hybrid application of FFT with a Bi-LSTM model, to predict electrical signals in DTC induction motors.

Despite the notable progress in the literature, several limitations must be addressed in current research endeavors. Notably, a substantial portion of studies has primarily fixated on forecasting stator currents, occasionally overlooking other pivotal signals such as rotor currents [36] or vice versa [37]. Additionally, the evaluation of model performance often revolves around a single evaluation metric, such as Mean Square Error (MSE), Mean Absolute Error (MAE), or Root Mean Square Error (RMSE), omitting a comprehensive visual juxtaposition of projected outcomes alongside actual data. This confined approach may hinder a comprehensive appraisal of the forecasting model’s precision and effectiveness. Table 1 presents the comparative analysis of these criteria.

## 3. Methodology

This section outlines the main methodology employed in our study, which encompasses data acquisition and modeling, our Bi-LSTM model’s architecture, the model training process, and the evaluation setting.

### 3.1. Modeling and Data Acquisition

**Figure 1:**
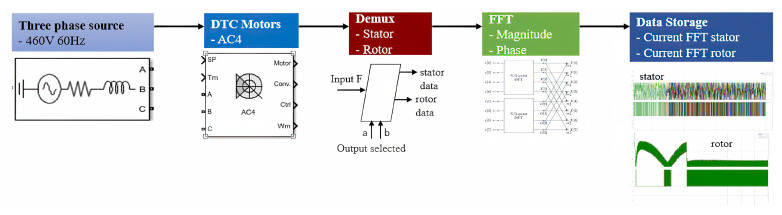
General concept of modeling and data acquisition.

**Figure 2:**
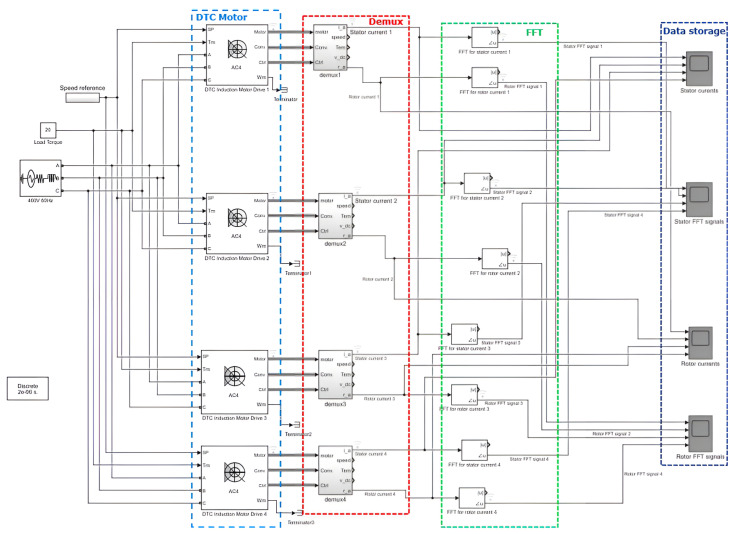
Detailed depiction of the approach involving four DTC induction motors.

This study utilized simulation data from several three-phase DTC induction motors. The simulation was conducted using Simulink software (MathWorks, Natick, MA, USA), a widely recognized simulation tool in electrical engineering [40]. The simulation generated a dataset comprising four multiple DTC induction motors, including stator currents and rotor currents. Appropriate preprocessing techniques were applied to ensure the dataset’s suitability for training the proposed model. Illustrated in Figure 1 is a conceptual overview of the approach for modeling and acquiring data. For a more intricate depiction, refer to Figure 2, which elaborates on the concept and includes four DTC induction motors.

In the context of DTC, demultiplexing (DEMUX) can be employed to separate different operating parameters of the motor, such as speed, torque, and current, into distinct output signals [41]. DTC is a technique used to manage the speed and torque of an induction motor by monitoring the currents flowing through the stator and making appropriate adjustments to the inverter’s switching patterns. Accurate and ongoing measurement of the stator currents and rotor position is crucial for the proper operation of DTC. Using DEMUX, the stator current and rotor position signals can be separated into independent output signals, enabling more precise control of the motor’s torque and speed [42].

#### 3.1.1. Mathematical Formulation of Currents in DTC Induction Motors

The AC4 motor drive, also called the DTC Induction Motor Drive block, is a commonly employed component within the Simscape Specialized Power Systems library [43]. Designed specifically for induction motors, this drive is an advanced control system providing direct torque and flux control capabilities. It encompasses closed-loop speed control and employs torque and flux controllers with hysteresis-band characteristics.

The induction motor block embodies a three-phase induction motor model. By employing the input voltages of the three phases, the block effectively regulates the individual currents of each phase. This enables precise control over the motor’s torque or speed dynamics [44]. The block employs equations formulated within a stationary rotor reference (dq) frame, encompassing the direct-axis (*d*-axis) and quadrature-axis (*q*-axis). Notably, the *d*-axis aligns with the a-axis, and all quantities in this rotor reference frame are referenced to the stator. In this work, our focus is placed on the stator and rotor *q*-and *d*-axis currents, and their calculations are outlined as follows:

(1) $$\left[\begin{array}{c} i_{sd}\\ i_{sq}\\ i_{rd}\\ i_{rq} \end{array}\right]=\left(\frac{1}{L_{m}^{2}-L_{r}L_{s}}\right)\left[\begin{array}{cccc} -L_{r} & 0 & L_{m} & 0\\ 0 & -L_{r} & 0 & L_{m}\\ L_{m} & 0 & -L_{s} & 0\\ 0 & L_{m} & 0 & -L_{s} \end{array}\right]\left[\begin{array}{c} \lambda_{sd}\\ \lambda_{sq}\\ \lambda_{rd}\\ \lambda_{rq} \end{array}\right]$$

In the aforementioned Equation (1): $i_{sq}$ and $i_{sd}$ represent the stator quadrature (*q*-axis) and direct (*d*-axis) currents, respectively, measured in amperes (A). $i_{rq}$ and $i_{rd}$ denote the rotor quadrature (*q*-axis) and direct (*d*-axis) currents, respectively, also measured in amperes (A). $L_{s}$ and $L_{r}$ stand for the stator and rotor inductances, respectively, measured in henrys (H). $L_{m}$ corresponds to the magnetizing inductance, measured in henrys (H). $\lambda_{sq}$ and $\lambda_{sd}$ represent the stator quadrature (*q*-axis) and direct (*d*-axis) flux, respectively, measured in webers (Wb). $\lambda_{rq}$ and $\lambda_{rd}$ denote the rotor quadrature (*q*-axis) and direct (*d*-axis) flux, respectively, also measured in webers (Wb).

In Equation (1), the concept of inductance in the context of DTC induction motors pertains to the inherent property of the motor’s coils to impede fluctuations in the current passing through them. Inductance can be mathematically determined using the following formula:

(2) $$L_{s}=L_{ls}+L_{m}$$

(3) $$L_{r}=L_{lr}+L_{m}$$

where $L_{ls}$ and $L_{lr}$ represent the stator and rotor leakage inductance, respectively, measured in henrys (H).

#### 3.1.2. DEMUX for DTC Induction Motor

Utilizing DEMUX in DTC offers multiple benefits. Firstly, it improves the performance of the motor by isolating different operating parameters, such as speed, torque, and current, and subsequently adjusting the control strategy based on these parameters. This enhances the efficiency and accuracy of the control system [45]. Moreover, demultiplexing the stator current and rotor position signals improves the accuracy of these measurements, thereby enhancing the overall performance of the DTC control system. It is crucial to emphasize the importance of accurately measuring the stator currents and rotor position for the proper functioning of DTC control. By incorporating demultiplexing techniques to separate the stator current and rotor position signals, the accuracy of these measurements can be improved, leading to enhanced performance of the DTC control system.

The DEMUX operation in DTC involves separating the stator current into its direct-axis and quadrature-axis components. This separation allows for independent control of these components, which is necessary for accurate torque and flux control. The DEMUX operation can be represented mathematically as:

(4) $$rsc\_sd=i_{sd}\times cos\left(\theta_{r}\right)$$

(5) $$rsc\_sq=i_{sq}\times sin\left(\theta_{r}\right)$$

where $i_{sd}$ and $i_{sq}$ represent the stator *d*-axis and *q*-axis in DTC induction motors component, $\theta_{r}$ denotes the rotor position, and rsc_sd and rsc_sq represent the *d*-axis and *q*-axis of the stator current components in DEMUX component, respectively.

The rotor current vector represents the current flowing through the rotor windings and is essential for accurate motor control. The rotor current vectors can be obtained using the following equations:

(6) $$rrc\_rd=rsc\_sd\times cos\left(\theta_{r}\right)-rsc\_sq\times sin\left(\theta_{r}\right)$$

(7) $$rrc\_rq=rsc\_sd\times sin\left(\theta_{r}\right)+rsc\_sq\times cos\left(\theta_{r}\right)$$

where rrc_rd and rrc_rq represent the rotor *d*-axis and *q*-axis currents in DEMUX component.

#### 3.1.3. FFT-Based Signal Processing

FFT is a mathematical technique that can be used to analyze the frequency components of a signal. The output signals of the DEMUX component for each induction motor can be used via FFT to extract relevant information about the frequency characteristics of the stator currents and rotor position data. By using FFT for the stator currents and rotor position signals, we can derive the frequency spectrum of the signals. It can assist in determining the dynamics of the motor and identify any unusual circumstances, such as stator winding flaws, rotor flaws, or other types of failures.

We can use mathematical equations and programming tools to calculate the FFT of the stator current and rotor current signals obtained from the DEMUX output of an induction motor. Here is a general procedure to calculate the FFT using mathematical equations:

- Prepare the input data: the stator current vector as rsc[n] and the rotor current vector as rrc[n], where *n* represents the discrete time index.
  (8) $$S\left[k\right]=\sum_{k=0}^{M-1}\left(rsc\left[n\right]\times exp\left(\frac{-i\times 2\times \pi \times k\times n}{M}\right)\right)$$
  (9) $$R\left[k\right]=\sum_{k=0}^{M-1}\left(rrc\left[n\right]\times exp\left(\frac{-i\times 2\times \pi \times k\times n}{M}\right)\right)$$
  where S[k] and R[k] are the complex-valued spectrum of the stator current and rotor current signals, respectively. rsc[n] and rrc[n] are the stator current and rotor current signals at discrete time index *n*. *M* is the length of the stator current and rotor current vectors.
- Extract the magnitude and phase information: To obtain the magnitude and phase information from the complex-valued FFT results, we can calculate the absolute value (magnitude) and phase angle of each FFT bin.
  (10) Mag_S[k]=abs(S[k])
  (11) Mag_R[k]=abs(R[k])
  (12) Pha_S[k]=atan2(Im(S[k]),Re(S[k]))
  (13) Pha_R[k]=atan2(Im(R[k]),Re(R[k]))
  where Mag_S[k] and Mag_R[k] are the magnitudes of the complex-valued FFT results for the stator current and rotor current signals, respectively. Pha_S[k] and Pha_R[k] are the phase angles of the complex-valued FFT results for the stator current and rotor current signals, respectively. Im(S[k]) and Re(R[k]) represent the imaginary and real parts of S[k], respectively. Im(R[k]) and Re(R[k]) represent the imaginary and real parts of R[k], respectively.

### 3.2. Bi-LSTM Model Architecture

**Figure 3:**
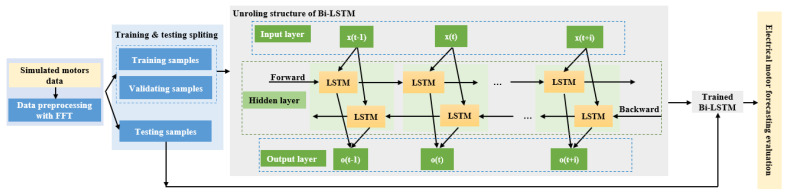
The architecture of the proposed method with unrolled structure of Bi-LSTM model.

In this section, we provide detail of our Bi-LSTM model approach. Figure 3 illustrates the general architecture of the proposed method, while the proposed model training and forecasting evaluation is pointed out in Algorithm 1.
**Algorithm 1** Model Training and Forecasting Evaluation**Require:** fsc, frc **Ensure:** Forecasting unseen stator FFT current seg_fsc,       Forecasting unseen rotor FFT current seg_frc1:Data preprocessing using MinMaxScale method:r,s←MinMaxScale(fsc,frc)2:Split preprocessed data into training and testing data with a ratio of 80:20, respectively// *For rotor data:*r_tr,s_tr←r[:len(r)×0.8],r[len(r)×0.8:]// *For stator data:*r_te,s_te←s[:len(s)×0.8],s[len(s)×0.8:]3:Create function split_IO() to split input and output from training and testing data(Xr_tr,yr_tr),(Xr_te,yr_te)←split_IO(r_tr,r_te)(Xs_tr,ys_tr),(Xs_te,ys_te)←split_IO(s_tr,s_te)4:Setup several hyperparameters of Bi-LSTM model:$n_{i},n_{h},n_{o}\leftarrow number\;of\;input,\;hidden,\;output\;nodes$op←trainingoptimizerloss←traininglossfunctionval←validatingsplitvaluecall←EarlyStoppingfunction5:**while** i≤epochs(ep)**do**6:    Create BiLSTM layer:    $BiLSTM\leftarrow LSTM(n_{i},n_{h},n_{o},n_{f},op,loss)$7:    Create fit() function to learn:     f_r←BiLSTM.fit(Xr_tr,yr_tr,ep,val,bat,call)     f_s←BiLSTM.fit(Xs_tr,ys_tr,ep,val,bat,call)8:**end while**9:Training evaluationmse_r,mse_s←f_r[loss],f_s[loss]10:Testing evaluationseg_frc←BiLSTM.prediction(Xr_te)seg_fsc←BiLSTM.prediction(Xs_te)11:Calculate RMSE and MAE losseserror_r←seg_frc−yr_teerror_s←seg_fsc−ys_termse_r←sqrt(mean((square(error_r))))rmse_s←sqrt(mean((square(error_s))))mae_r←mean(abs(error_r))mae_s←mean(abs(error_s))12:**return** seg_frc,seg_fsc

During preprocessing, we employ the MinMaxScaler function from the scikit-learn library to preprocess the data. This step is crucial as normalizing the data has been shown to enhance the performance of neural networks. The data transformation process involves three key steps. Firstly, we fit the scaler using the available training data, which entails estimating the minimum and maximum observable values based on the training data. Subsequently, we apply the scaler to the training data. Finally, we apply the same scaler to the test data. The MinMaxScaler technique is widely utilized for normalizing data, enabling the scaling of dataset values to a predefined range, often between 0 and 1. The mathematical formulation of the MinMaxScaler for FFT-transformed current data is as follows:

(14) $$S_{i}\_scaled=\frac{S_{i}-S\_min}{S\_max-S\_min}$$

where $S_{i}$, S_min, S_max are an individual sample, the minimum value, the maximum value from the stator FFT current data *S*, $S_{i}\_scaled$ is the scaled value of $S_{i}$ in the range [0,1].

(15) $$R_{i}\_scaled=\frac{R_{i}-R\_min}{R\_max-R\_min}$$

where $R_{i}$, R_min, R_max are an individual sample, the minimum value, and the maximum value from the rotor FFT current data *R*; $R_{i}\_scaled$ is the scaled value of $R_{i}$ in the range [0,1].

The Bi-LSTM architecture is an RNN type that excels at capturing long-term dependencies in sequential data. In contrast to traditional RNNs, which process data in a single direction, Bi-LSTMs simultaneously process input data in both forward and backward directions. This bidirectional processing enables the network to effectively gather information from past and future contexts. Our proposed Bi-LSTM model comprises multiple layers of Bi-LSTM units, each followed by a dense layer with a linear activation function. The input to the model is a sequence of historical electrical signal data, and the output is the predicted value for the next time step. To train the Bi-LSTM model, we employ the backpropagation algorithm with the Mean Squared Error (MSE) loss function. We utilize the Adam optimizer, a widely-used algorithm for training Deep Neural Networks (DNNs) for optimization. The outputs from both directions of the Bi-LSTM are combined through concatenation and passed through a fully connected layer, followed by an activation function, to generate the final output.

The forward LSTM can be represented as:

(16) $$h_{t}^{f}=\sigma_{f}\left(W_{f}x_{t}+U_{f}h_{t-1}^{f}+b_{f}\right)$$

(17) $$\begin{array}{c} c_{t}^{f}=f_{f}\left(W_{f}x_{t}+U_{f}h_{t-1}^{f}+b_{f}\right)⊙c_{t-1}^{f}+i_{f}\left(W_{f}x_{t}+U_{f}h_{t-1}^{f}+b_{f}\right)⊙\tanh\left(W_{f}x_{t}+U_{f}h_{t-1}^{f}+b_{f}\right) \end{array}$$

where $x_{t}$ is the input at time step *t* (we assume that $x_{t}$ represents for stator FFT current *S* or rotor FFT current *R*), $h_{t}^{f}$ is the hidden state of the forward LSTM at time step *t*, $c_{t}^{f}$ is the cell state of the forward LSTM at time step *t*, $W_{f}$, $U_{f}$, and $b_{f}$ are the weights and biases of the forward LSTM, $\sigma_{f}$ is the sigmoid activation function, $f_{f}$ is the forget gate, $i_{f}$ is the input gate, and ⊙ denotes element-wise multiplication.

The backward LSTM can be represented as:

(18) $$h_{t}^{b}=\sigma_{b}\left(W_{b}x_{t}+U_{b}h_{t+1}^{b}+b_{b}\right)$$

(19) $$\begin{array}{c} c_{t}^{b}=f_{b}\left(W_{b}x_{t}+U_{b}h_{t+1}^{b}+b_{b}\right)⊙c_{t+1}^{b}+i_{b}\left(W_{b}x_{t}+U_{b}h_{t+1}^{b}+b_{b}\right)⊙\tanh\left(W_{b}x_{t}+U_{b}h_{t+1}^{b}+b_{b}\right) \end{array}$$

where $h_{t}^{b}$ and $c_{t}^{b}$ are the hidden state and cell state of the backward LSTM at time step *t*, respectively, $W_{b}$, $U_{b}$, and $b_{b}$ are the weights and biases of the backward LSTM, $\sigma_{b}$ is the sigmoid activation function, $f_{b}$ is the forget gate, $i_{b}$ is the input gate, and ⊙ denotes element-wise multiplication.

The output of the Bi-LSTM model can be computed as follows:

(20) $$y_{t}=\sigma \left(W_{o}\left[h_{t}^{f};h_{t}^{b}\right]+b_{o}\right)$$

where $\left[h_{t}^{f};h_{t}^{b}\right]$ is the concatenation of the forward and backward hidden states, $W_{o}$ and $b_{o}$ are the weights and biases of the output layer, and σ is the activation function.

### 3.3. Model Training and Evaluation Setting

The optimal architecture of the network was determined through systematic experimentation and iterative refinement. The aim was to identify the configuration that best aligned with the intricacies of the problem at hand, thereby maximizing forecasting accuracy. The process involved exploring various combinations of hyperparameters, including the number of LSTM layers, the number of neurons per layer, the dropout rate, and the choice of activation functions.

To facilitate this exploration, we harnessed a simulation dataset containing electrical signal data from three-phase Direct Torque Control (DTC) induction motors. This dataset was meticulously collected and preprocessed to ensure its suitability for training and evaluation. During experimentation, the dataset was randomly partitioned into a training set (80%) and a testing set (20%) to provide a robust assessment of model performance.

To optimize the training process, we utilized the Adam optimizer with a learning rate 0.001. The training and validation procedures were conducted in batches of 64 samples and over a maximum of 100 epochs. Notably, the choice of the batch size and epoch limit was motivated by a balance between computational efficiency and model convergence.

To establish the best network architecture, we examined a range of hyperparameters. The number of LSTM layers and neurons per layer was adjusted to evaluate their impact on performance. Additionally, the dropout rate was explored to mitigate overfitting tendencies. Activation functions were also scrutinized for their role in enabling the network to capture complex relationships within the data.

The iterative experimentation process enabled us to compare the model’s performance across various configurations systematically. The criteria for determining the optimal architecture were based on the lowest validation loss and enhanced predictive accuracy. The specific hyperparameters that yielded the best results for our proposed Bi-LSTM model are documented in Table 2.

Particularly, the EarlyStopping method plays a pivotal role in identifying the juncture at which the validation loss either stabilizes or begins to ascend. This critical point signifies the optimal convergence of the model, striking a delicate equilibrium between the pitfalls of underfitting and overfitting. In our experimentation, we harnessed the power of the EarlyStopping method, seamlessly integrated through the Python Keras library, to ascertain the ideal number of epochs for our training regimen. This method operates by continuously tracking a designated metric throughout the training process and intervenes when the monitored metric ceases to exhibit further enhancement. This strategic intervention identifies the precise moment of optimal convergence, thus facilitating the determination of the most suitable number of epochs for our comprehensive training and validation analyses.

When evaluating loss, $mse_{r}$ and $mse_{s}$ signify the losses on the rotor and stator validation sets. Meanwhile, ep corresponds to the number of epochs. The EarlyStopping mechanism endeavors to minimize the validation loss across the epochs. Consequently, the optimal number of epochs for rotor data ($ep_{r}$) and stator data ($ep_{s}$) can be mathematically represented as:

(21) $$ep_{r}=argmin_{ep}mse_{r}\left(ep\right)$$

(22) $$ep_{s}=argmin_{ep}mse_{s}\left(ep\right)$$

In order to evaluate the effectiveness of our approached model, three widely used evaluation metrics, specifically MSE, RMSE, and MAE, are utilized. These evaluation metrics act as indicators of the model’s precision. MSE is particularly useful in identifying outlier predictions with significant errors, as it emphasizes these errors due to the squaring operation in its calculation (refer to (23)). Since the squaring operation ensures that MSE is always non-negative, it provides a means to evaluate the model’s performance without considering the direction of errors. RMSE, on the other hand, is a widely used metric that quantifies the difference between predicted and actual values (refer to (24)). While RMSE is sensitive to outliers, MAE is less affected by them and still provides valuable insights into prediction accuracy. MAE, which differs slightly in definition from MSE, involves taking the absolute difference between model predictions and ground truth and averaging these absolute differences across the entire dataset (refer to (25)). The three measures are defined as follows:

(23) $$MSE=\frac{1}{n}\sum_{i=1}^{n}{\left(y_{i}^{t}-y_{i}^{p}\right)}^{2}$$

(24) $$RMSE=\sqrt{\frac{\sum_{i=1}^{n}y_{i}^{t}-y_{i}^{p}}{n}}$$

(25) $$MAE=\frac{1}{n}\sum_{i=1}^{n}\left|y_{i}^{t}-y_{i}^{p}\right|$$

where *n* denotes the total number of samples, and $y^{t}$ and $y^{p}$ represent the actual and predicted motor signal data at the *i* th second time data, respectively.

Furthermore, in order to comprehensively assess the performance of our proposed Bi-LSTM model, we conducted a thorough evaluation involving other state-of-the-art time series forecasting models. This comparative analysis encompassed well-established models, including RNN (Recurrent Neural Network) [46], LSTM (Long Short-Term Memory) [47], and GRU (Gated Recurrent Unit) [48], all trained and evaluated using the same dataset and evaluation criteria.

LSTM networks represent a variant of RNNs that leverage specialized memory cells designed to retain and retrieve information over extended periods. These memory cells employ gating mechanisms to regulate the flow of information in and out of the cell. Conversely, GRU models offer a simplified rendition of LSTMs, utilizing a solitary update gate to control memory cell information flow, in contrast to LSTMs’ three-gate configuration. This streamlined design renders GRUs more training-efficient and quicker in execution, though their capacity to retain and access prolonged dependencies may differ from LSTMs.

Both LSTMs and GRUs aim to tackle the challenge of vanishing gradients that can impede learning in traditional RNNs. This challenge arises when weight gradients diminish substantially, hindering effective learning. LSTMs and GRUs have found applications across diverse domains, such as language translation, speech recognition, and time series forecasting.

By comparing the results of these models against our method’s outcomes, we sought to accentuate the practical advantages and advancements realized through our innovative approach. This comprehensive comparison highlights the strengths of our proposed Bi-LSTM model and underscores its significance in addressing complex time series forecasting tasks.

## 4. Results and Discussion

This section delves into a comprehensive discussion of our experimental findings. Initially, we examine the impact of employing FFT analysis on the extracted stator and rotor current data. Subsequently, we assess the performance of our proposed method by utilizing various loss metrics, including MSE, for both the training and validation processes and RMSE and MAE for the testing process, on the stator FFT current and rotor FFT current. Additionally, we validate the effectiveness of our proposed Bi-LSTM approach by contrasting its accuracy with that of the contemporary GRU time series forecasting model. Lastly, we present compelling evidence of the superior forecasting performance of our proposed method compared to the GRU model when applied to the same experimental data.

### 4.1. Effect of Stator and Rotor FFT Current Data

In this section, we investigate applying the FFT method for extracting valuable information from DTC induction motors’ stator and rotor current data. The FFT method is a widely used technique in signal processing that converts time-domain signals into the frequency domain. By leveraging this transformation, we can gain deeper insights into the spectral characteristics and frequency components present in the motor currents.

The effectiveness of employing the FFT method in different domains for signal analysis has been well established. For example, the FFT technique has been utilized in power systems to examine power quality concerns such as harmonic distortion and voltage fluctuations [49]. Similarly, in vibration analysis, the FFT method has been widely used to identify and analyze the frequency components of mechanical vibrations, aiding in detecting faults and anomalies in rotating machinery [50].

**Figure 4:**
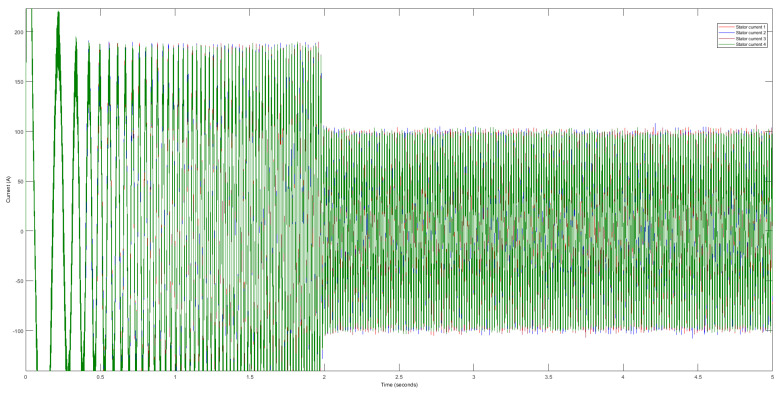
Raw data of current stator signals.

**Figure 5:**
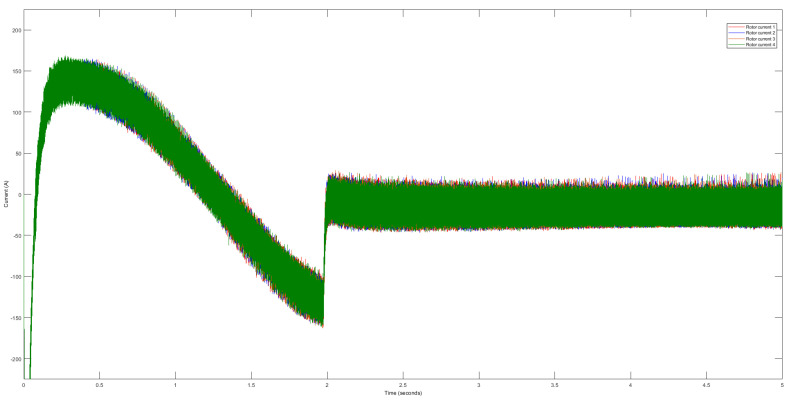
Raw data of current rotor signals.

First, we analyze the raw data obtained from four identical DTC induction motors. We plot the stator and rotor current data on a single graph to provide a comprehensive overview. However, the raw data exhibit inherent variability, presenting interpretation and further analysis challenges. Upon closer examination, we observe minor differences among the raw stator signals acquired from the four motors, as illustrated in Figure 4. Similarly, we note slight disparities in the rotor signals, as depicted in Figure 5. However, discerning or distinguishing these signals with the naked eye proves to be quite arduous. It poses substantial difficulties when identifying or forecasting patterns within these seemingly similar motor signal datasets.

**Figure 6:**
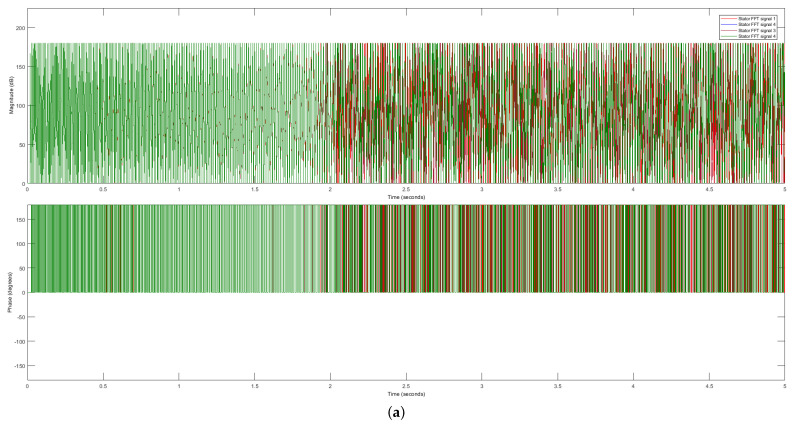

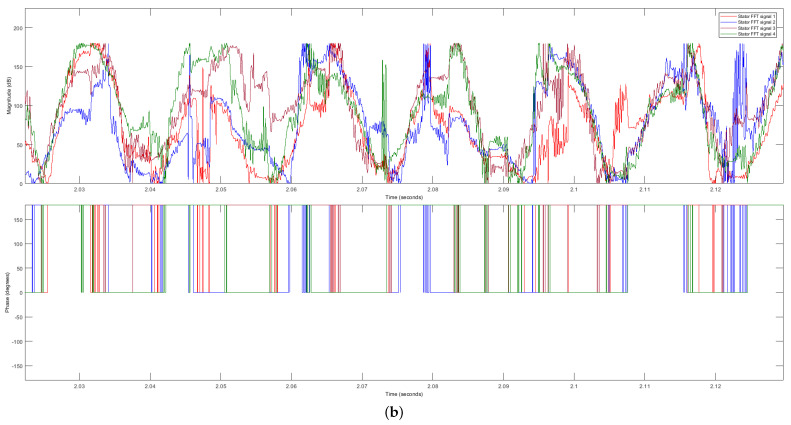
Stator FFT signals. (**a**) Magnitude and phase of stator FFT signals. (**b**) Magnified view of the magnitude and phase of stator FFT signals.

**Figure 7:**
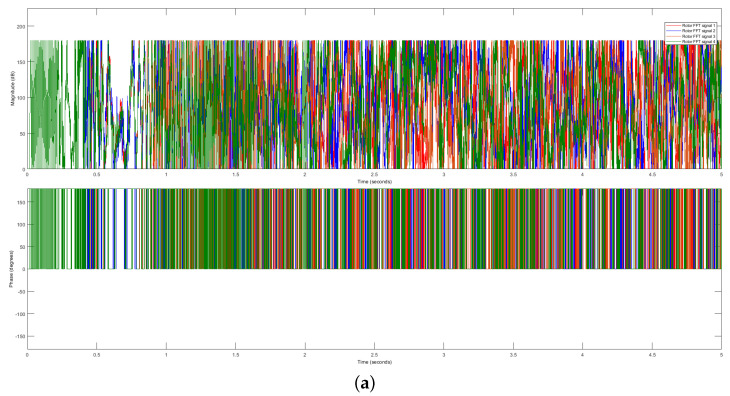

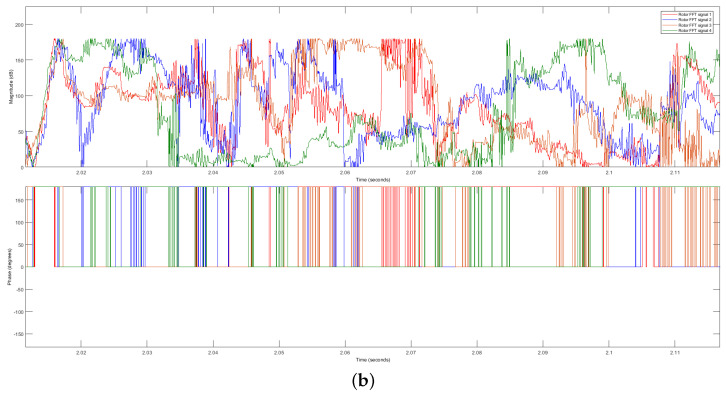
Rotor FFT signals. (**a**) Magnitude and phase of the rotor’s FFT signals. (**b**) Enlarged view of the magnitude and phase of the rotor’s FFT signals.

We employ the FFT extraction method to address the challenges posed by the variability in the raw data. This technique transforms the raw time-domain signals into frequency-domain signals, yielding magnitude and phase data. The resulting stator and rotor FFT signals are presented in Figure 6 and Figure 7, respectively. These frequency-domain signals exhibit distinct magnitudes and phases, providing valuable information for further analysis and processing, such as forecasting or classification tasks.

Comparing the raw data with the extracted frequency-domain data reveals significant differences among the same four motor signals. This disparity is demonstrated in Figure 6 (stator FFT current data) and Figure 7 (rotor FFT current data). In order to provide a more detailed examination of the stator and rotor FFT current data, we zoom in on the plots, as illustrated in Figure 6b and Figure 7b, respectively.

The observed dissimilarities in the FFT representation of the four DTC induction motor signals highlight the effectiveness of the FFT method in capturing and distinguishing important features. Consequently, we utilize the stator and rotor FFT currents to evaluate our proposed approach.

### 4.2. Comparing Forecasting Performance: Bi-LSTM Model and Other Forecasting Models

Utilizing simulated data, we evaluate our proposed Bi-LSTM model’s capability to forecast electrical signals from DTC induction motors. Our assessment encompasses examining FFT impact on stator and rotor current data, employing diverse performance metrics. These metrics include MSE loss for training and validation assessment and RMSE and MAE losses for testing evaluation.

Furthermore, we undertake a comparative analysis by juxtaposing the performance of our proposed approach, which leverages a Bi-LSTM model for time-series forecasting against state-of-the-art (SOTA) and widely recognized forecasting models. This comparison includes established models, including RNN, LSTM, and GRU.

This comprehensive evaluation highlights the strengths and advancements of our proposed Bi-LSTM model while demonstrating its superiority over well-known forecasting alternatives.

#### 4.2.1. Loss Metrics Measurement

In order to assess the efficacy of the proposed approach, we computed MSE loss for both the training and validation datasets, as outlined in Table 3 for stator data and Table 4 for rotor data. In both the training and validation phases, the Bi-LSTM model showcased superior performance compared to the LSTM and GRU models for both stator and rotor data. Additionally, our findings revealed a slightly enhanced accuracy of the proposed model in predicting the stator FFT currents over the rotor FFT currents. Conversely, the RNN model displayed the highest MSE loss, indicating its inability to forecast motor data effectively.

Further evaluations of the proposed Bi-LSTM model were conducted by assessing MAE and RMSE on the testing dataset, with the outcomes presented in Table 5 for stator data and Table 6. Similar to the unfavorable MSE outcome observed with the RNN model, the MAE and RMSE of the RNN model also exhibited considerably high values. These findings underscore that the proposed Bi-LSTM model outperforms the standard LSTM and GRU models regarding forecasting precision.

Specifically, a comparative analysis was performed on the predicted stator FFT current and rotor FFT current of the Bi-LSTM, LSTM, and GRU models against the actual stator FFT current and actual rotor FFT current, followed by the calculation of MSE and RMSE values. The notably lower values of MSE and RMSE provide clear evidence that the proposed model can precisely forecast the rotor FFT current.

#### 4.2.2. Forecasting Evaluation Illustration

**Figure 8:**
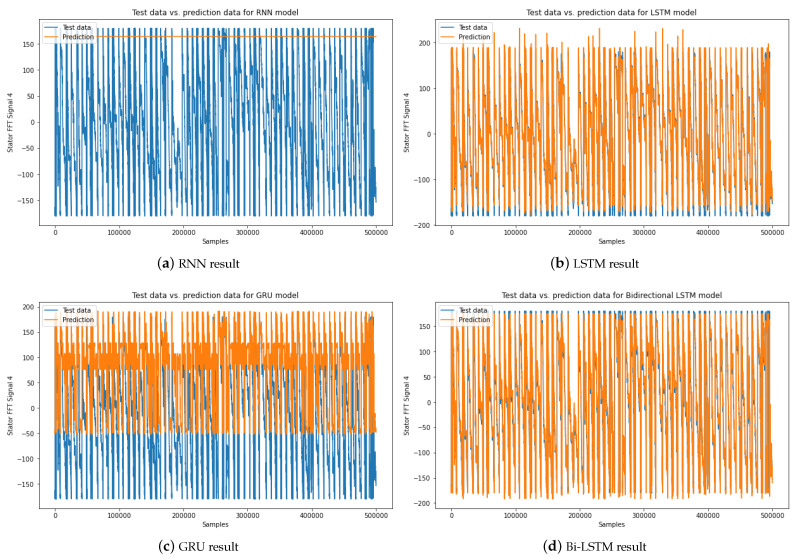
Comparing stator FFT signal forecasting results: (**a**) Predicted stator FFT signal of motor 4 using the RNN model, (**b**) Predicted stator FFT signal of motor 3 using the LSTM model, (**c**) Predicted stator FFT signal of motor 3 using the GRU model, and (**d**) Predicted stator FFT signal of motor 3 using the Bi-LSTM model.

**Figure 9:**
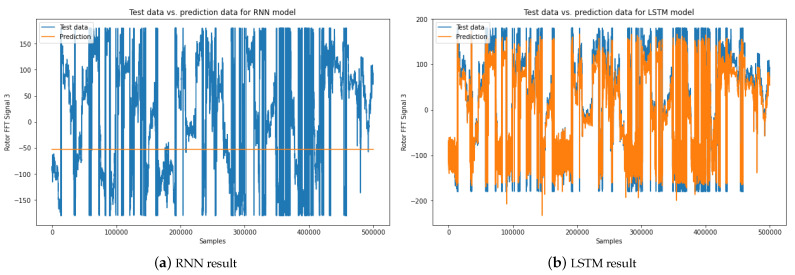

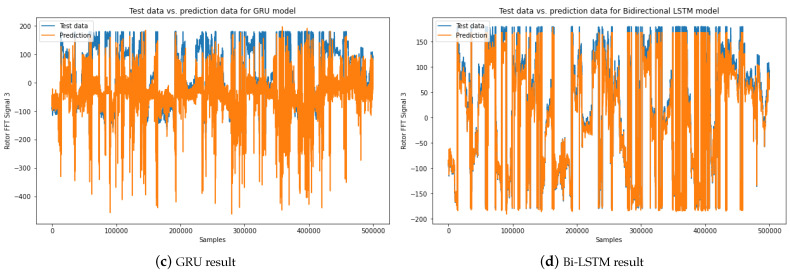
Comparing rotor FFT signal forecasting results: (**a**) Predicted rotor FFT signal of motor 3 using the RNN model, (**b**) Predicted rotor FFT signal of motor 3 using the LSTM model, (**c**) Predicted rotor FFT signal of motor 3 using the GRU model, and (**d**) Predicted rotor FFT signal of motor 3 using the Bi-LSTM model.

The forecasting evaluation outcomes for both the Bi-LSTM model and several alternative forecasting models (RNN, LSTM, GRU) are depicted in Figure 8 and Figure 9. Specifically, Figure 8 provides a visual representation of the stator FFT forecasting results of motor 4. The results distinctly demonstrate the superior performance of the Bi-LSTM model compared to the other models. The RNN model’s inability to forecast the stator FFT current signal is evidenced in Figure 8a. The RNN model yielded the poorest forecasting results, primarily due to its vulnerability to the vanishing gradient problem during extended training periods [47]. Consequently, this model struggles to manage temporal dependencies effectively. In contrast, the LSTM model achieves a reasonably accurate forecasting result, as illustrated in Figure 8b. Notably, the predicted stator FFT current signals generated by the Bi-LSTM model exhibit a notably closer alignment with the actual signals. This alignment signifies the model’s enhanced accuracy and precision, outperforming the GRU model’s result and slightly surpassing the LSTM model’s result.

Similarly, Figure 9 underscores the significant superiority of the Bi-LSTM model in predicting rotor FFT data. While the RNN model faces challenges in predicting rotor FFT current data, as evident in Figure 9c, the Bi-LSTM model distinguishes itself by yielding reduced noise and heightened accuracy in comparison to the GRU and LSTM models’ outcomes.

These compelling findings accentuate the exceptional forecasting performance of the Bi-LSTM model in accurately predicting electrical signals from three-phase DTC induction motors.

The assessment metrics, encompassing MSE, RMSE, and MAE, collectively corroborate the model’s efficacy by showcasing its ability to achieve notably low error values. This outcome underscores the model’s proficiency in accurately forecasting stator and rotor FFT currents. Furthermore, the Bi-LSTM model shines particularly bright in predicting the rotor FFT current of the motor. This observation further accentuates its excellence in capturing intricate patterns and complex trends present within the data. The model’s robust performance is a testament to its advanced capabilities in deciphering and forecasting electrical signals, thereby contributing to enhanced precision and reliability in practical forecasting scenarios.

In summary, the comprehensive comparative evaluation against conventional RNN, LSTM, and GRU models notably highlights the outstanding capabilities of the proposed Bi-LSTM model. It consistently demonstrates superior accuracy and substantially reduces prediction errors when considering stator and rotor data.

## 5. Conclusions

This study has introduced a novel approach to forecast electrical signals within three-phase DTC induction motors accurately. By effectively harnessing the potential of the transformed FFT from simulation data and integrating it with the Bi-LSTM network for time series forecasting, we have successfully captured the intricate dynamics inherent in multiple induction motors. As a result, our work highlights the approach’s remarkable accuracy in predicting electrical signals, outperforming conventional RNN, LSTM, and GRU models. It underscores the effectiveness of the Bi-LSTM model in capturing temporal dependencies present in the data.

The outcomes of this study provide valuable insights into the refinement of precise forecasting models for predicting the electrical behavior of DTC induction motors. These insights, in turn, play a pivotal role in enhancing motor performance, elevating condition monitoring capabilities and optimizing operational efficiency. We recognize the importance of aligning our research with practical industry applications so that our future research endeavors will encompass a real-world case study. This study will serve as a tangible exemplification, showcasing our proposed approach’s practical implementation and effectiveness within shipboard energy systems.

## Figures and Tables

**Table 1:** Comparison of existing studies with our work based on two criteria: simulated motor data and forecasting evaluation metrics.

Study	Simulated Stator Data	Simulated Rotor Data	MSE	MAE	RMSE	Forecasting Visualization
[36]	✓	✗	✓	✗	✗	✗
[37]	✓	✓	✗	✗	✗	✗
[38]	✗	✓	✗	✓	✗	✓
[39]	✓	✓	✓	✓	✗	✓
**Ours**	✓	✓	✓	✓	✓	✓

Legend: ✓ means included, ✗ means not included.

**Table 2:** Hyperparameter setting for Bi-LSTM model and other forecasting models.

Hyperparameters	Variable	Value
Number of training samples	no_training_samples	1,999,970
Number of testing samples	no_testing_sample	499,970
Number of time steps	no_timesteps	30
Number of input neurons	no_input_node (ni)	64
Number of hidden neurons	no_hidden_node (nh)	64
Number of output neurons	no_output_node (no)	1
Number of features	no_features	1
Dropout layer	dropout	0.2
Training optimizer	optimizer (op)	Adam
Batch size	batch_size (bat)	16
Training shuffle	shuffle	false
Number of epochs	epochs(ep)	100
Validation ratio	val	0.2

**Table 3:** MSE results of Bi-LSTM model and other forecasting models for training and validating evaluation on stator data.

Evaluation Process	Model	Stator Data			
		Motor 1	Motor 2	Motor 3	Motor 4
Train	RNN	0.0176	0.0174	0.0185	0.0192
	LSTM	0.0007	0.0006	0.0007	0.0009
	GRU	0.0012	0.0007	0.0008	0.0137
	**Bi-LSTM**	**0.0003**	**0.0004**	**0.0006**	**0.0003**
Validate	RNN	0.3088	0.6026	0.2686	0.2820
	LSTM	0.0009	0.0020	0.0006	0.0006
	GRU	0.0006	0.0019	**0.0005**	0.0628
	**Bi-LSTM**	**0.0005**	**0.0013**	0.0012	**0.0005**

**Table 4:** MSE results of Bi-LSTM model and other forecasting models for training and validating evaluation on rotor data.

Evaluation Process	Model	Rotor Data			
		Motor 1	Motor 2	Motor 3	Motor 4
Train	RNN	0.0210	0.0209	0.0207	0.0048
	LSTM	0.001	0.0009	0.0014	0.0014
	GRU	0.0011	0.0028	0.0045	0.0011
	**Bi-LSTM**	**0.0006**	**0.0005**	**0.0006**	**0.0006**
Validate	RNN	0.0872	0.0868	0.0862	0.0328
	LSTM	0.0015	0.0016	0.0044	0.0043
	GRU	0.0019	0.00267	0.0465	0.0017
	**Bi-LSTM**	**0.0006**	**0.0008**	**0.0015**	**0.0005**

**Table 5:** MAE and RMSE results of Bi-LSTM model and other forecasting models for testing evaluation on stator data.

Evaluation Metric	Model	Stator Data			
		Motor 1	Motor 2	Motor 3	Motor 4
MAE	RNN	163.5519	255.4206	164.3720	163.6676
	LSTM	90.3153	102.7191	89.9001	85.9685
	GRU	88.8639	102.4355	92.3747	104.9523
	**Bi-LSTM**	**86.3565**	**101.4268**	**87.1624**	**95.8002**
RMSE	RNN	163.5522	255.4208	164.3722	163.6680
	LSTM	103.6535	115.1428	103.7457	99.9042
	GRU	102.7566	114.9634	105.7504	105.8054
	**Bi-LSTM**	**99.9575**	**114.9324**	**100.4124**	**104.9523**

**Table 6:** MAE and RMSE results of Bi-LSTM model and other forecasting models for testing evaluation on rotor data.

Evaluation Metric	Model	Stator Data			
		Motor 1	Motor 2	Motor 3	Motor 4
MAE	RNN	154.6887	154.2273	153.5426	135.8195
	LSTM	84.0248	84.9345	74.3362	83.3043
	GRU	90.4460	86.7933	83.8774	90.4395
	**Bi-LSTM**	**81.8216**	**84.8299**	**46.1538**	**82.7156**
RMSE	RNN	154.6895	154.2281	153.5433	139.9584
	LSTM	96.3886	96.1901	83.2497	101.6299
	GRU	102.7570	98.8687	96.3640	102.6000
	**Bi-LSTM**	**93.3045**	**95.1969**	**60.1075**	**94.5194**

## Data Availability

Not applicable.

## Abbreviations

The following abbreviations are used in this manuscript:

ANNs	Artificial Neural Networks
Bi-LSTM	Bidirectional Long Short-Term Memory
CNNs	Convolutional Neural Networks
DEMUX	Demultiplexing
DNNs	Deep Neural Networks
DTC	Direct Torque Control
DL	Deep Learning
FFT	Fast Fourier Transform
GRU	Gated Recurrent Unit
MAE	Mean Absolute Error
MSE	Mean Squared Error
LSTM	Long Short-Term Memory
RNN	Recurrent Neural Network
RMSE	Root Mean Squared Error
